# Reduced tonic inhibition in striatal output neurons from Huntington mice due to loss of astrocytic GABA release through GAT-3

**DOI:** 10.3389/fncir.2013.00188

**Published:** 2013-11-26

**Authors:** Anna M. Wójtowicz, Anton Dvorzhak, Marcus Semtner, Rosemarie Grantyn

**Affiliations:** ^1^Cluster of Excellence NeuroCure, University Medicine CharitéBerlin, Germany; ^2^Department of Experimental Neurology, University Medicine CharitéBerlin, Germany; ^3^RNA Editing and Hyperexcitability Disorders Group, Department of Neuroscience, Max Delbrück Center for Molecular MedicineBerlin, Germany

**Keywords:** GABAergic synaptic transmission, astrocyte, GAT-3, ambient GABA, GABA(A) receptor, GABA(B) receptor, presynaptic depression, Huntington's disease

## Abstract

The extracellular concentration of the two main neurotransmitters glutamate and GABA is low but not negligible which enables a number of tonic actions. The effects of ambient GABA vary in a region-, cell-type, and age-dependent manner and can serve as indicators of disease-related alterations. Here we explored the tonic inhibitory actions of GABA in Huntington's disease (HD). HD is a devastating neurodegenerative disorder caused by a mutation in the huntingtin gene. Whole cell patch clamp recordings from striatal output neurons (SONs) in slices from adult wild type mice and two mouse models of HD (Z_Q175_KI homozygotes or R6/2 heterozygotes) revealed an HD-related reduction of the GABA(A) receptor-mediated tonic chloride current (I_Tonic(GABA)_) along with signs of reduced GABA(B) receptor-mediated presynaptic depression of synaptic GABA release. About half of I_Tonic(GABA)_ depended on tetrodotoxin-sensitive synaptic GABA release, but the remaining current was still lower in HD. Both in WT and HD, I_Tonic(GABA)_ was more prominent during the first 4 h after preparing the slices, when astrocytes but not neurons exhibited a transient depolarization. All further tests were performed within 1–4 h *in vitro*. Experiments with SNAP5114, a blocker of the astrocytic GABA transporter GAT-3, suggest that in WT but not HD GAT-3 operated in the releasing mode. Application of a transportable substrate for glutamate transporters (D-aspartate 0.1–1 mM) restored the non-synaptic GABA release in slices from HD mice. I_Tonic(GABA)_ was also rescued by applying the hyperagonist gaboxadol (0.33 μM). The results lead to the hypothesis that lesion-induced astrocyte depolarization facilitates non-synaptic release of GABA through GAT-3. However, the capacity of depolarized astrocytes to provide GABA for tonic inhibition is strongly reduced in HD.

## Introduction

In contrast to the low-affinity GABA(A) receptors in the postsynaptic density, extrasynaptic GABA(A) receptors populating the somato-dendritic membrane of mature neurons display sufficient sensitivity to report the presence of GABA at submicromolar concentrations (Semyanov et al., 2004; Farrant and Nusser, 2005; Glykys and Mody, 2007a; Brickley and Mody, 2012). In addition, low concentrations of GABA in the environment of synaptic terminals are detected by presynaptic GABA(B) receptors (Le Feuvre et al., 1997; Kirmse and Kirischuk, 2006; Mapelli et al., 2009; Dvorzhak et al., 2010; Laviv et al., 2010). Both signaling pathways may interact for homeostatic adjustment of synaptic *vs.* non-synaptic GABAergic input (Mody, 2005; Connelly et al., 2013).

Tonic GABA actions vary strongly in a region—as well as cell-type-specific manner (Semyanov et al., 2003; Song et al., 2011). In the rodent striatum, where GABA-synthesizing cells constitute the vast majority of the neuron population, tonic chloride currents via extrasynaptic GABA(A) receptors (termed I_Tonic(GABA)_) were consistently recorded as depolarizing shifts in the baseline currents in response to GABA(A) receptor block (Ade et al., 2008; Janssen et al., 2009; Santhakumar et al., 2010; Cepeda et al., 2013). Changes in vesicular transmitter release after bath-application of a specific GABA(B) receptor-blocker present evidence for tonic presynaptic GABA effects, at least under condition of reduced GABA uptake (Kirmse et al., 2009). The indicators of tonic extrasynaptic and presynaptic GABA actions can mutually validate each other, although the presynaptic depression is probably less sensitive (Kirmse et al., 2008).

It is obvious that the strength of tonic inhibition will not only depend on the characteristics of the respective receptors (Scimemi et al., 2005; Ransom et al., 2010) and the amount of “spillover” after vesicular GABA release (Glykys and Mody, 2007b) but also on the distribution and transport of GABA from and into the extracellular space (Wu et al., 2007; Ransom et al., 2013). In the present study, the focus will be placed on the role of the astrocytic GABA transporter GAT-3 as a potential source of non-synaptic GABA release (Yoon et al., 2012).

A comprehensive description of the structure, cellular distribution and function of GABA transporters (GATs) can be found in (Borden, 1996; Dalby, 2003; Richerson and Wu, 2003; Jin et al., 2011a). Four different GATs are known, with somewhat confusing nomenclature in mice *vs.* rat. The expression of GAT-1 and GAT-3 is brain-specific, where GAT-1 protein is preferentially localized in neurons, notably in their presynaptic terminals (Radian et al., 1990), and GAT-3 is preferentially localized on the processes of astrocytes (Minelli et al., 1996; Ribak et al., 1996).

As other neurotransmitter transporters, GABA transporters can reverse their transport direction (Wu et al., 2007). However, the conditions necessary for reversal may vary in dependence of the given stoichiometry. While a glutamate uptake cycle through the major astrocytic transporter GLT-1 (EAAT-2) is associated with an inward move of three Na^+^ and one proton and an outward move of one K^+^, a GABA uptake cycle through GAT-3 only co-transports two Na^+^ and one Cl^−^. Thus, the driving forces at resting membrane potentials are much lower for the GABA transport. Reversal may therefore easily occur under a variety of physiological as well as pathophysiological conditions.

In general, one can expect that excessive excitation leading to membrane depolarization, loss of potassium, and elevation of intracellular sodium levels will increase the likelihood that sites of GABA uptake are converted into sites of non-synaptic GABA release (Richerson and Wu, 2003). At the same time, glutamate transporters might continue to operate in the uptake mode. Thus, within a range of cellular perturbations, opponent glutamate and GABA transport may form a basis of neuroprotection, as recently suggested by Heja et al. (2012). These authors presented evidence that high activity of astrocytic glutamate uptake facilitates non-synaptic GABA release through GAT-3. Their hypothesis also predicts that, due to co-localization of GLT-1 and GAT-3, a failure in astrocytic glutamate uptake would affect the neuronal response to non-synaptic GABA release from astrocytes.

A devastating neurological condition associated with insufficiency of astrocytic glutamate uptake is Huntington's disease (HD). HD belongs to the group of inherited polyglutamine disorders that derive from a CAG triplet repeat expansion. The pathologically expanded tract of glutamines is toxic and causes neurodegeneration, especially in the striatum. At earlier stages of the disease, the afflicted individuals suffer from emotional and cognitive disturbances, but also from uncoordinated movements. This state (Ross and Tabrizi, 2011; Ha and Fung, 2012) is also known as chorea. Striatal neurons expressing a mutant form of huntingtin are characterized by hyperexcitability (Dvorzhak et al., 2013) and pathological spontaneous action potential generation (Miller et al., 2011). Several studies have suggested that in the HD striatum reduced glutamate uptake through GLT-1 may impair the capacity of astrocytes to support normal levels of neuronal activity (Lievens et al., 2001; Behrens et al., 2002; Miller et al., 2008; Faideau et al., 2010; Petr et al., 2013), but it is not yet known as to what extent tonic GABA effects, in general, and GAT-3 function, in particular, were altered in symptomatic HD.

In the present study, we have used two different mouse models of HD to quantify the possible changes in tonic extrasynaptic and presynaptic GABA actions. The results presented here will lead to the conclusion that I_Tonic(GABA)_ and tonic GABA(B) receptor-mediated depression of synaptic GABA release are less potent in HD because the HD-afflicted astrocytes have a reduced capacity to release GABA via GAT-3.

## Materials and methods

### Ethical approval

The present experiments were performed in fully adult (315–450 days) wild-type mice from a colony of Z-Q175-KI provided by the CHDI (“Cure Huntington's Disease Initiative”) foundation and R6/2 mice provided by Charles River GmbH Germany. Every precaution was taken to minimize stress and to reduce the number of animals used in each series of experiments. The work described here has been carried out in accordance with the EU Directive *2010/63/EU* for animal experiments and complies with the requirements for manuscripts submitted to biomedical journals. The work was registered at the Office of Health Protection and Technical Safety of the regional government Berlin (Landesamt für Arbeitsschutz, Gesundheitsschutz und Technische Sicherheit Berlin, T0448/12).

### Transgenic mice

The Z-Q175-KI mouse line was created by Cerebricon Ltd/Charles River Labs, Kuopio, Finland and CHDI Fndn. Inc., Princeton, NJ, and provided by the Jackson Labs, Bar Harbor, USA (ME #370437). It originated from the Q140 knock-in (KI) mouse described by Menalled et al. (2003) and express a chimeric mouse/human exon 1 *mhtt*. The R6/2 mouse line (B6CBA-Tg(HDexon1)62Gpb/3J, Jackson Labs, Bar Harbor, ME #006494) was derived from the transgenic mouse line generated by Mangiarini et al. (1996). The mice were genotyped by PCR and analyzed for CAG length. Experiments were either performed in Q175 homozygotes or in R6/2 heterozygotes and their respective wild-type siblings. We shall refer to the normal wild-type mice as “WT” and to mice carrying a mutant form of huntingtin as “HD mice” or just “HD.” For more information on the mice see Dvorzhak et al. (2013). Briefly, average CAG length was 120 ± 0.2 (range = 114–127, *n* = 137) in R6/2 and 184.6 ± 0.7 (range = 172–195, *n* = 68) in Q175. The average age was 78.0 ± 1.1 d, *n* = 91 vs. HD 75.0 ± 1.2 d, *n* = 77 (n.s.) in R6/2 and 400.3 ± 2.6 d, *n* = 54 vs. 405.6 ± 4.9 d, *n* = 68 (n.s.) in Q175. All of the Q175 mice were older than 1 year. Considering the absence of sex- and age-dependent differences in the tested parameters we have accordingly pooled the data from the WT and HD cohorts.

### Slice preparation

The animals were deeply anesthetized by inhalation of a mixture of isoflurane and carbogen (95% O_2_ and 5% CO_2_) and transcardially perfused with 60 ml of ice-cold (~4°C) saline containing (in mM): 125 choline chloride, 2 KCl, 1.25 NaH_2_PO_4_, 25 NaHCO_3_, 0.5 CaCl_2_, 7 MgCl_2_, 10 glucose, 0.5 CaCl_2_, 7 MgCl_2_ (pH 7.25, 305 mosmol/l). The 1 year and older Q175 mice were initially prepared in the same solution, but in a second series of experiments (Figures 4, 5) the Q175 mice were transcardially perfused with ice-cold saline before removing the brains from the skull. The choline-based perfusion solution was supplemented with 5 μM glutathione, 500 μM Na pyruvate, 2.8 mM ascorbic acid, and 1 μM of MK-801. The brain was removed quickly (~1 min), separated into two hemispheres and transferred into ice-cold oxygenated saline. Sagittal slices of 300 μm were prepared with a vibrating microtome (Integraslice 7550PSDS, Campden Instruments Ltd., Loughborough, UK) and then maintained for at least 1 h in artificial cerebrospinal fluid (ACSF) that contained (in mM): 125 NaCl, 2 KCl, 1.25 NaH_2_PO_4_, 25 NaHCO_3_, 2 CaCl_2_, 1 MgCl_2_, 10 glucose, 0.5 pyruvic acid, 0.005 glutathione, and 2.8 ascorbic acid(pH 7.35, 303 mosmol/l).

### Solutions and chemicals

During experiments, 10 μM DNQX and 50 μM DL-2-amino-5-phosphonopentanoic acid (APV) were added to the ACSF to block glutamatergic currents. The remaining postsynaptic currents were completely blocked by bicuculline methiodide (BMI, 25 μM) or SR-95531 (gabazine, 10 μM), but not by strychnine (30 μM), indicating their GABAergic nature. The following pharmacological agents were obtained from Tocris (Bristol, UK) and applied at indicated concentrations: gaboxadol or 4,5,6,7-tetrahydroisoxazolo[5,4-c]pyridin-3-ol hydrochloride (THIP, 0.1-1 μM), (2*S*)-3-(1*S*)-1-(3,4-dichlorophenyl)ethyl]amino-2-hydroxypropyl](phenylmethyl)phosphinic acid (CGP55845, CGP, 1 μM), LY341495 (40 μM and 0.1 μM), 1-[2-[*tris*(4-methoxyphenyl)methoxy]ethyl]-(*S*)-3-piperidinecarboxylic acid ((S)-SNAP5114, 40 μM). Tetrodotoxin (TTX, 1 μM) was obtained from Alomone Labs (Jerusalem, Israel). All other chemicals were from Sigma-Aldrich (Munich, Germany), including 1-[2-[[(diphenylmethylene)imino]oxy]ethyl]-1,2,5,6-tetrahydro-3-pyridinecarboxylic acid hydrochloride (NO-711, 10 μM), D-aspartic acid monosodium salt (D-Asp, 0.1-1 mM). Other concentrations are given in the Results section.

### Patch clamp recording

For electrophysiological tests, slices were submerged into a perfusion chamber with a constant flow of oxygenated ACSF. The flow rate was set to 1–2 ml min^−1^. Temperature during the recordings was maintained at 26–27°C. Preceding experiments at various maintenance temperature (range = 23–30°C) showed that under the given conditions slices from animals >1 year were best maintained at this temperature, the quality criterion being the level of neuronal membrane potentials at break-in, in addition to the appearance of the slices. In HD mice, one could see debris from degenerating axons in the globus pallidus and substantia nigra suggesting some degree of neurodegeneration.

Pipette resistance was 3-6 MOhm when filled with the following saline (in mM): 150 KCl, 5 NaCl, 0.5 CaCl_2_, 5 EGTA, 25 HEPES, 2 MgATP, 0.3 GTP (for recording tonic chloride currents) and 100 potassium gluconate, 50 KCl, 5 NaCl, 0.5 CaCl_2_, 5 EGTA, 25 HEPES, 2 MgATP, 0.3 GTP (for recording IPSCs). The calculated equilibrium potentials for chloride [E_Cl^–^_] were 4 and −21 mV, respectively.

Electrophysiological signals were acquired using an EPC-8 amplifier (List, Darmstadt, Germany), a 16-bit AD/DA board (ITC-16, HEKA Elektronik, Lambrecht, Germany) and the software TIDA 4.11 (HEKA Elektronik, Lambrecht, Germany). The signals were sampled at a rate of 10 kHz and filtered at 3 kHz. Liquid junction potentials were not corrected. In neurons, the holding potential was set to −70 mV which was close to the resting membrane potential recorded in these cells in the current clamp mode immediately after break-in. Access resistance was monitored by applying pulses of −10 mV. Cell capacitance and access resistance values were obtained by fitting a mono-exponential function to the capacitance transients. Only recordings with a series resistance below 30 MOhm were accepted (in typical cases series resistance amounted to 15-20 MOhm). Series resistance compensation was not applied. Cells exhibiting more than a 20% change in the access resistance during an experiment were discarded. Resting membrane potentials of neurons and suphorhodamine-labeled astrocytes were measured immediately after break-in.

## Identification of astrocytes

Protoplasmic astrocytes were identified by staining with sulforhodamine 101 (SR101) (Nimmerjahn et al., 2004). The original protocol was modified to optimize the labeling in slices from adult and, in case of HD, rather sick mice. Briefly, 30 min after preparation slices were transferred into oxygenized ACSF containing 6 μM SR101 and maintained at 37°C for 10 min. After this the slices were washed and stored for at least 40 min in standard ACSF at room temperature. Astrocyte staining was visualized under fluorescence optics using an excitation wavelength of 573 nm controlled by a monochromator system (polychrome V) from Till Photonics (Gräfelfing, Germany). The dichroic mirror (QMAX/DI580LP) and emission filter (QMAX/EM600-650) were from Omega Optical, Brattleboro, VT, USA.

### Electrical stimulation and recording of unitary EPSCs

Unitary evoked excitatory postsynaptic currents (eEPSCs) were elicited by intrastriatal microstimulation via a glass pipette filled with ACSF as described before (Dvorzhak et al., 2013). Briefly, glass pipettes filled with ACSF had a resistance of about 10 MOhm and were moved in the dorsal striatum in the vicinity of the recorded neuron until the recording pipette detected a postsynaptic response. Sometimes neurons were filled with Lucifer Yellow which helped to avoid positions that could lead to direct depolarization of dendrites. An isolated stimulation unit was used to generate rectangular electrical pulses. Pulse duration was set at 0.5 ms. Pulse intensity was adjusted to activate a synaptic response at minimal intensity and with distinct threshold. Stimulation was accepted as unitary if the following criteria were satisfied: (1) eIPSC latency remained stable (<20% fluctuations), (2) lowering stimulus intensity by 20% resulted in a complete failure of eIPSCs, (3) an increase in stimulus intensity by 20% neither changed mean eIPSC amplitude nor eIPSC shape, (4) there was no contamination by glutamatergic synaptic input. Typical current intensities required for unitary stimulation ranged between 0.4 and 0.6 μA. Paired pulses were delivered at an inter-stimulus-interval of 50 ms at a repetition frequency of 0.1–0.2 Hz to allow for full recovery of transmitter release after paired pulse stimulation at an interval of 50 ms.

Although there is no proof that only 1 axon had been stimulated in any given case, we regard it as highly probable that the responses induced by minimal stimulation were indeed “unitary eIPSCs,” i.e., responses to activation of just one presynaptic neuron. For the sake of simplicity, evoked IPSCs will in the following be referred to as eIPSCs, assuming that in the vast majority of cases we have dealt with unitary connections.

### Data evaluation and statistics

All data was evaluated off-line using TIDA 4.11 (HEKA Elektronik, Lambrecht, Germany), IgorPro6.0 (WaveMetrics, Lake Oswego, OR, USA), Prism 6.01 (Graphpad, San Diego, CA, USA), SPSS 21 (SPSS GmbH Software, Munich, Germany). I_Tonic(GABA)_ was defined as the difference of baseline currents in the absence and presence of BMI (20 μM). The size of I_Tonic(GABA)_was verified by plotting all-points histograms for 30 s periods immediately before and during BMI application using a custom-written macro in IgorPro6.0 (WaveMetrics, Lake Oswego, OR, USA). Gaussian functions were fitted to the part of the distribution not skewed by synaptic events, and I_Tonic(GABA)_ was determined as the distance between the two amplitude peaks.

The quantitative results are presented as mean ± s.e.m. The error bars in the figures indicate s.e.m. Data points and means from HD mice are presented with gray fill. The numbers in brackets are the number of tested neurons. Normality of data distributions was evaluated by the Kolmogorov–Smirnov or the Shapiro–Wilk test. Differences between means were determined by paired or unpaired *t*-test (normally distributed data) and Wilcoxon matched-pairs signed rank test or Mann–Whitney test (not normally distributed data). Bonferroni correction was implemented in case of multiple comparison tests. One-Way ANOVA (normally distributed data) or Kruskal–Wallis statistics (not normally distributed data) was used for differences between 3 groups of data from different cells. Two-Way ANOVA with *post-hoc* Bonferroni correction was performed for experiments in the classical 2 × 2 design, with 2 independent factors (genotype and treatment). Fitting of regression curves to data points was performed on the basis of the Spearman test. The asterisks in the figures indicate the following: ^*^p < 0.05, ^**^p < 0.01, and ^***^p < 0.001.

## Results

### The GABA(A) receptor-mediated tonic chloride current and the GABA(B) receptor-mediated depression of synaptic GABA release as reporters of ambient GABA concentration in the murine striatum

**Figure 1:**
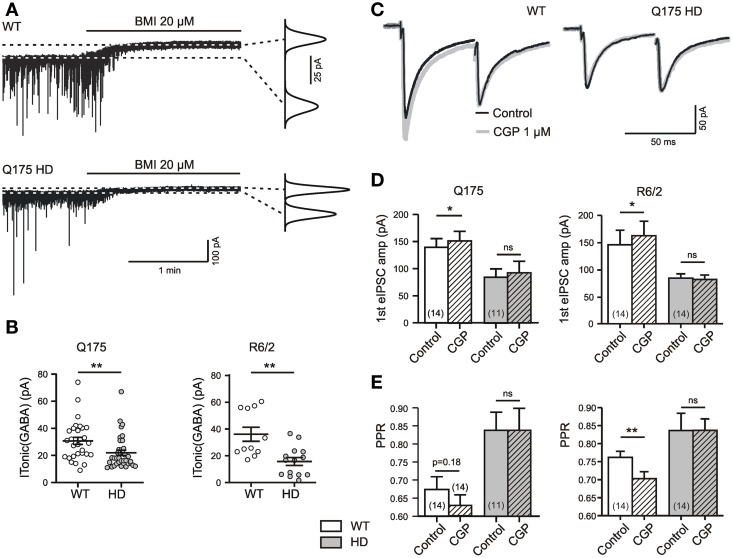
**HD-related differences in tonic GABA action in the striatum. (A)** Specimen records of I_Tonic(GABA)_ from SONs in slices from 1 year old WT and Q175 HD. Experiments in the presence of DNQX (10 μM), APV (50 μM), LY341495 (40 μM); no K^+^ channel block. Graphs on the right: Gaussian fits to all-points histograms derived from 30 s recording periods under control conditions and in the presence of BMI. The difference between the peaks of the fitting curves represents the amplitude of I_Tonic(GABA)_ as plotted in **(B)**. **(B)** I_Tonic(GABA)_ obtained from WT (empty symbols) and HD mice (filled symbols). Note similar results in the two mouse models of HD. Significance levels according to Mann–Whitney-test. **(C)** Sample traces to illustrate the differential effects of GABA(B) unblocking with CGP (1 μM) in WT and HD. Records under similar conditions as in panel **(E)**. **(D,E)** Quantification of results for eIPSC amplitude and PPR. Note lack of CGP effect in HD mice and similar results from Q175 and R6/2 WT. Significance levels according to paired *t*-tests. The asterisks denote the following: ^*^p < 0.05, ^**^p < 0.01, and ^***^p < 0.001.

Extrasynaptic GABA(A) receptors and presynaptic GABA(B) receptors display high affinities for GABA, the respective EC_50_ values in slice preparations being 0.1–0.25 μM (Dvorzhak et al., 2010) and 1.5–5 μM (Le Feuvre et al., 1997). They are therefore well-suited to indicate the presence of extracellular GABA concentrations ([GABA]_ec_) in the lower micromolar range. Moreover, the combined use of the two indicators helps to mutually validate the obtained results. Figure 1 illustrates this experimental approach when applied to WT and HD SONs.

The GABA(A) receptor-mediated I_Tonic(GABA)_ was defined as the baseline current shift recorded during the application of 20 μM of BMI (Figure 1A). The experiments were performed at a high intracellular chloride concentration without addition of potassium channel blockers and rendered similar results in WT SONs from the Q175 and the R6/2 colony (Figure 1B).

Tonic GABA(B) receptor-mediated effects were determined in a similar manner, but by bath application of the GABA antagonist CGP at a concentration of 1 μM. This concentration is sufficient to block saturating GABA(B) effects of GABA (Le Feuvre et al., 1997). CGP-induced changes in the paired pulse ratio (PPR), in addition to changes in the amplitude of unitary evoked IPSCs (eIPSCs), indicate a GABA(B)-dependent depression of synaptic GABA release (see Dvorzhak et al., 2013, for details). Recordings of eIPSCs from adult WT SONs (Figure 1C) illustrate that synaptic GABA release is indeed reduced by tonically active GABA(B) receptors. The typical finding after CGP administration was an increase of the first eIPSC and a decrease of the PPR (Figures 1C–E). Again, similar results were obtained in WT from the Q175 and R6/2 colonies.

Together, these results are in line with previous evidence suggesting a role of ambient GABA in the regulation of activity levels in the rodent striatum (Ade et al., 2008; Kirmse et al., 2008; Janssen et al., 2009; Santhakumar et al., 2010; Cepeda et al., 2013).

### HD-related differences in tonic GABA actions

Under standard recording conditions, the tonic actions of GABA were weaker in HD. Again, this applies to the GABA(A) receptor-mediated I_Tonic(GABA)_ (1A, B) and to the GABA(B) receptor-mediated presynaptic depression of synaptic GABA release (Figures 1C–E). Moreover, results obtained in Q175 were reproduced in R6/2 (Table 1).

**Table 1:** **Characteristics of I_(Tonic)GABA_ in two mouse models of HD**.

	**WT**			**HD**			***p***
	**Mean**	**±*SE***	***N***	**Mean**	**±*SE***	***N***	
**Q175 SERIES 1**							
Standard	30.7	2.6	30	21.9	2.1	37	0.0028
+ TTX 1 μM	17.9	2.1	15	11.7	1.2	23	0.0016
+ TTX + NO-711 10 μM	43.2	6.9	12	68.0	8.3	18	0.0306
**Q175 SERIES 2**							
+ TTX 1 μM	34.1	4.2	12	16.9	1.5	13	0.0003
+ TTX + SNAP5114 40 μM	22.9	3.2	13	35.0	4.2	7	0.0771
+ TTX + D-Asp 0.1 mM	32.7	5.4	15	38.0	4.5	18	n.s.
**R6/2**							
Standard	36.1	5.2	11	15.7	3.0	14	0.0018
+ TTX 1 μM	16.1	3.2	9	4.7	2.2	9	0.0078
+ TTX + NO-711 10 μM	55.4	10.6	11	83.8	11.0	11	0.0648
+ TTX + D-Asp 0.1 mM	12.4	1.6	11	15.9	3.9	13	n.s.

The significance levels of the differences between means from WT and HD apply to Mann–Whitney tests.

**Figure 2:**
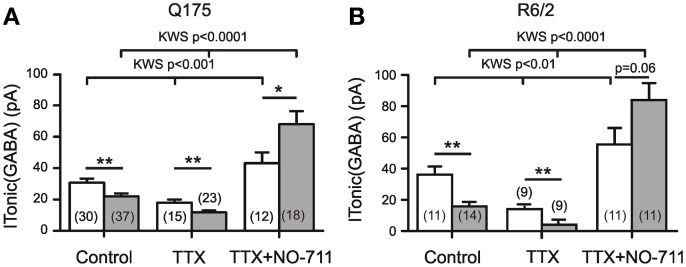
**Influence of synaptic release and GAT-1 activity on I_Tonic(GABA)_.** Recording conditions and supplements as in Figure 1A. **(A,B)** Results from two different types of HD mice. TTX (1 μM) reduces I_Tonic(GABA)_ without abolishing the HD-related difference. Block of GAT-1 with NO-711 (10 μM) increases I_Tonic(GABA)_. Note significantly stronger effect of NO-711 in HD mice (filled bars). In both graphs, the data sets of the 6 columns were independent, not paired. In the 3 groups for Control, TTX, and TTX+NO-711 the means from WT vs. HD were compared according to Mann–Whitney tests (see Table 1 for more details). In addition, we have performed Kruskal–Wallis statistics (KWS) to compare the differences between Control, TTX, and TTX plus NO-711 in WT and HD. The respective values were as follows. Q175 WT: KWS = 15.23, *p* < 0.001; Q175 HD: KWS = 50.91, *p* < 0.0001. R6/2 WT: KWS = 12.40, *p* < 0.01; R6/2 HD: KWS = 23.94, *p* < 0.0001. The asterisks denote: ^*^p < 0.05, ^**^p < 0.01, and ^***^p < 0.001.

The following experiments were aimed at clarifying the origin of the HD-related differences in tonic GABA actions. First we addressed the question as to what extent action potential-(AP)-dependent synaptic GABA release contributed to the ambient GABA level that maintains I_Tonic(GABA)_. The results of AP block with TTX (1 μM) are presented in Figures 2A,B. It can be seen that in both HD models, TTX significantly reduced the amplitude of I_Tonic(GABA)_ suggesting that synaptically released GABA is a major determinant of the ambient GABA concentration. However, the differences between WT and HD SONs remained. We therefore concluded that extrasynaptic release of GABA can play a role in the regulation of I_Tonic(GABA)_.

Next we examined the effects of NO-711 (10 μM), a blocker of the predominantly neuronal GABA transporter GAT-1. Block of GAT-1 was found to increase I_Tonic(GABA)_, particularly in the HD mice, suggesting that the neuronal GABA transport operates in the uptake mode. The results also indicate that in HD mice, the tonic GABA effect is not limited by the availability of extrasynaptic receptors. Again, the results were similar in Q175 and R6/2 (Figures 2A,B, Table 1).

### A possible dependence of I_Tonic(GABA)_ on the state of astrocytes

Since all results were obtained from acutely prepared brain slices, we had to address the caveat that the obtained data were influenced by the preparation procedure, recording conditions and/or the recovery dynamics after slicing. Indeed, years ago we have reported results from immature rat (Meier et al., 2003) indicating that slicing and tissue maintenance *in vitro* stimulates a PKC- and Ca-dependent process of GABAergic synaptogenesis. One has to assume that the acute slice approach includes an acute response to lesion. We therefore considered the possibility that the perturbation of the tissue and the recovery dynamics after preparing the slices might differentially affect the size of I_tonic(GABA)_ in WT and HD mice. But what is the cellular basis of this differential reaction?

**Figure 3:**
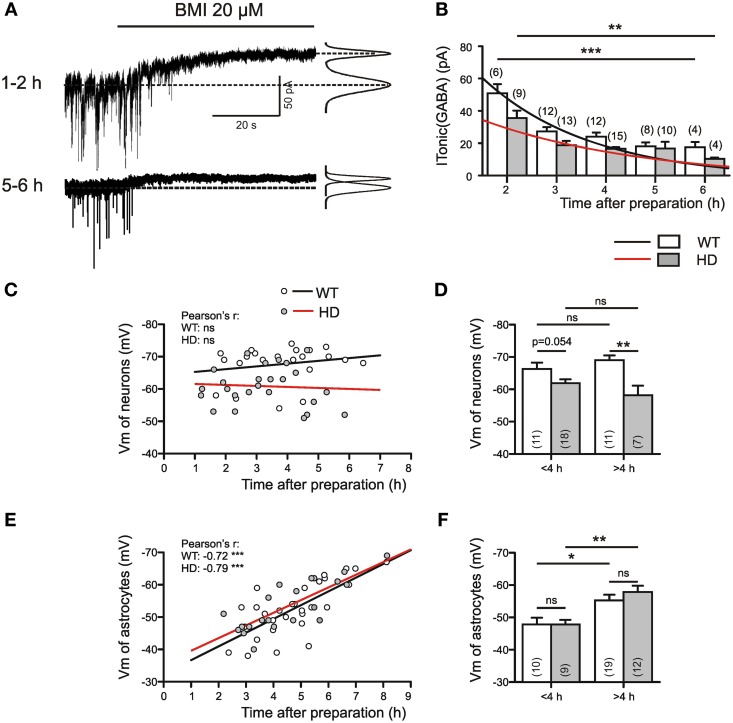
**I_Tonic(GABA)_ in WT and Q175 HOMs at different times after slice preparation. (A)** Sample traces from 2 different WT SONs from the Q175 colony; age: 55 weeks. Experiment in the presence of DNQX (10 μM), APV (50 μM), LY341495 (40 μM); no K channel block; *V*_*h*_ = −70 mV, *E*_Cl_ = 4 mV. On the right: Gaussian fits to all-points histograms derived from 30 s recording periods under control conditions and in the presence of BMI. The difference between the peaks of the fitting curves represents the amplitude of I_Tonic(GABA)_ as plotted in **(B)**. **(B)** Average values of I_Tonic(GABA)_ obtained from WT (empty bars) and Q175 HD (filled bars) at variable time after preparation. The differences between 2 and 6 h values were significant for WT at *p* = 0.0007 and for HD at *p* = 0.005 (Mann–Whitney test). **(C,D)** Plots of neuronal membrane potential against time *in vitro*. Note absence of time-dependency in WT or HD **(C)**, but significantly lower average membrane potentials in the >4 h HD group. **(E,F)** Plots of astrocytic membrane potentials against time *in vitro*. Note that with time astrocytic membrane potentials shift toward more negative values **(E)**, without any difference between WT and HD **(F)**. The significance levels in **(D,F)** refer to Mann–Whitney tests. The asterisks denote the following: ^*^p < 0.05, ^**^p < 0.01, and ^***^p < 0.001.

As a very first step toward answering this question we examined the time dependency of I_Tonic(GABA)_ in Q175 WT and HOMs (Figures 3A,B). It turned out that both in WT and HD SONs I_Tonic(GABA)_ was larger during the first 4 h after cutting the slices. Next we examined the membrane potentials (V_m_) of neurons (Figures 3C,D) and astrocytes (Figures 3E,F) and found relatively stable neuronal V_m_ (albeit lower in HD), but a very pronounced time dependency of astrocytic V_m_. Thus, high amplitudes of I_Tonic(GABA)_ coincided in time with astrocyte depolarization. This finding is consistent with two conclusions: (i) lesion-induced astrocyte depolarization promotes the reversal of the astrocytic GABA transporter GAT-3; (ii) in HD, an additional mechanism attenuates the non-synaptic release of GABA from astrocytes. In any case, the HD-related differences in I_Tonic(GABA)_ cannot be attributed to differences in the electrical driving force for astrocytic GABA release.

### Dependency of tonic GABA actions on the astrocytic GABA transporter GAT-3

**Figure 4:**
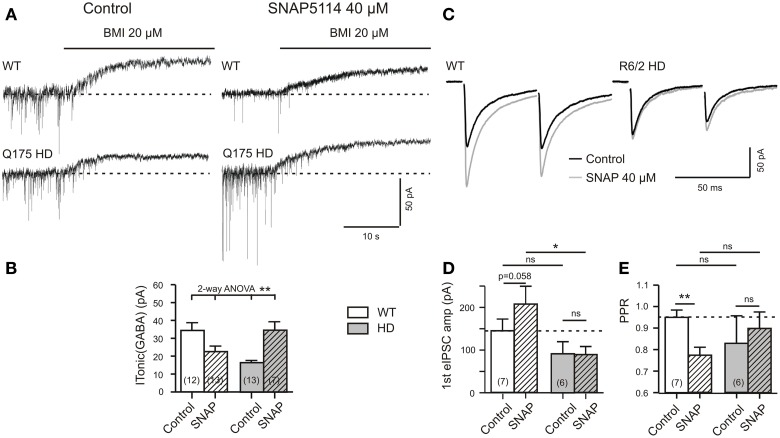
**HD-related differences in the effect of SNAP5114 on I_Tonic(GABA)_ and presynaptic depression of evoked GABA release. (A)** Sample traces of I_Tonic(GABA)_ from a Q175 WT and HD SONs. Recordings in the presence of TTX (1 μM), CGP (1 μM), DNQX (10 μM), APV (50 μM), and LY341495 (40 μM). Note opposite effects of GAT-3 block with SNAP5114 (40 μM) in WT and HD. **(B)** Quantification of results from Q175 WT and HD. Significance level according to 2-Way ANOVA with subsequent Bonferroni correction for four comparisons. Normality was verified by the Kolmogorov–Smirnov test. The ANOVA table gave the following results. Interaction: *F*_(1, 41)_ = 14.51, *p* = 0.0005; Genotype: *F*_(1, 41)_ = 0.0683, *p* = 0.7951, n.s.; Treatment with SNAP5114: *F*_(1, 41)_ = 0.4032, *p* = 0.5290, n.s. **(C–E)** Experiments in R6/2 WT and HD. Additions: APV 50 μM, DNQX 20 μM, LY341494 40 μM. **(C)** Records of averaged eIPSCs under the indicated conditions. Note that in WT, but not HD, SNAP5114 removed a presynaptic depressant effect, as indicated by the significant decrease of the PPR **(E)**. Significance levels according to paired *t*-tests (comparison Control vs. SNAP) and unpaired *t*-tests (comparison WT vs. HD). The asterisks denote the following: ^*^p < 0.05, ^**^p < 0.01, and ^***^p < 0.001.

To further validate the role of the astrocytic GABA transporter GAT-3 slices were for at least 7 min incubated in the GAT-3-specific blocker SNAP5114 (40 μM). All recordings were performed in Q175, within the first 4 h after slicing and in the presence of TTX. Figures 4A,B presents the results of the experiments with SNAP5114. Interestingly, SNAP5114 had opposite effects in WT and HD SONs. In WT, SNAP tended to reduce I_Tonic(GABA)_ while SNAP5114 treatment of HD slices resulted in a significant increase. These results support the conclusion that in WT, but not in HD mice, GAT-3 contributes to I_Tonic(GABA)_ and operates in the releasing mode. The weaker tonic GABA action in HD may then reflect the loss of GAT-3-dependent GABA release from astrocytes.

Would this hypothesis be supported in the experiments with CGP? Figures 4C–E presents the results. Indeed, SNAP5114 increased the amplitude of the 1st eIPSC and decreased the PPR, as it is to be expected when GAT-3 is blocked in the releasing mode. HD preparations lacked this effect.

### Dependency of tonic GABA actions on glutamate transporter activity

The HD-related deficiency of astrocytic GABA release under condition of strong depolarization could reflect the weakness of astrocytic glutamate uptake and the resultant change in the local sodium gradient as a cause of GAT-3 reversal. The experiments of Heja and colleagues (Heja et al., 2009, 2012) illuminated the dependency of the astrocytic GABA transporter GAT-3 on the state of glutamate transporters. In the rodent hippocampus, GAT-3 and GLT-1 (EAAT-2) are co-localized (Heja et al., 2012) and therefore exhibit a significant overlap of the respective intracellular [Na^+^] domains. The Na^+^ influx associated with glutamate uptake by GLT-1 was regarded as a prerequisite for the calcium-independent GABA release by GAT-3 from astrocytes. Astrocytic depolarization and astrocytic glutamate uptake may jointly drive the non-synaptic release of GABA from astrocytes. While the former condition was present both in WT and in HD, the latter was absent in HD.

**Figure 5:**
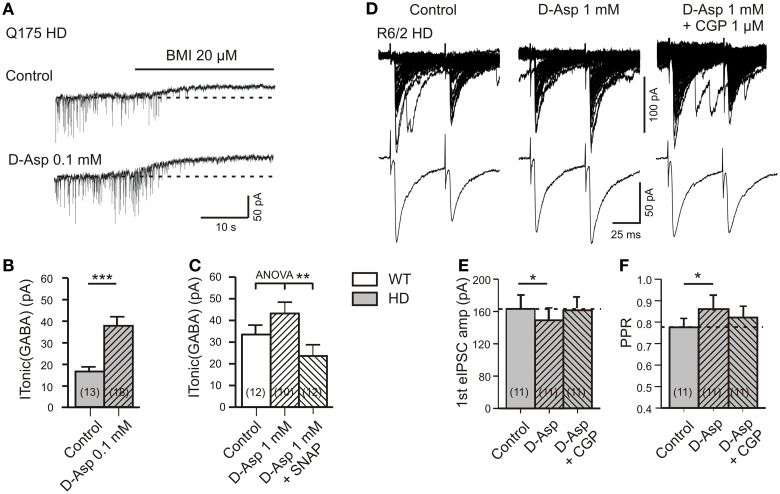
**Effects of D-aspartate on I_Tonic(GABA)_ and GABA(B) receptor-mediated presynaptic depression of synaptic GABA release. (A–C)** Experiment with Q175 WT and HD. Recordings in the presence of DNQX (10 μM), APV (50 μM), and LY341494 (40 μM). **(A)** Sample traces from a Q175 HD SON in the absence and presence of D-aspartate (0.1 mM). Note the larger I_Tonic(GABA)_ after 10 min treatment of HD slices with D-aspartate. **(B)** Quantification of the results from Q175 HD mice. Significance level according to Mann–Whitney test. **(C)** I_Tonic(GABA)_ of WT SONs treated with 1 mM D-aspartate for 5 min. Note the highly significant decrease in the presence of SNAP5115 (40 μM). Significance level according to One-Way ANOVA with *post-hoc* Bonferroni correction [*F*_(2, 31)_ = 6.603, *p* = 0.0041]. The data sets represented in the three columns are independent, not matched. **(D–F)** eIPSCs in R6/2 WT and HD SONs in the presence of APV (50 μM), DNQX (20 μM), and LY341494 (40 μM). **(D)** Records of averaged eIPSCs from one and the same HD SON under the indicated conditions. Note the rescue of tonic GABA(B)-mediated depression of synaptic GABA release in HD after treatment of 1 mM D-aspartate for 5 min. **(E,F)** Quantification of the results from HD SONs tested for changes in eIPSC amplitude **(E)** and PPR **(F)** in the presence of D-aspartate and D-aspartate plus CGP. The indicated differences between Control and D-aspartate reflect the amount of tonic presynaptic depression of synaptic GABA release according to Wilcoxon's matched-pairs signed rank test after Bonferroni correction for multiple comparison. ^*^p < 0.05, ^**^p < 0.01, and ^***^p < 0.001.

If correct, stimulation of glutamate uptake with D-aspartate might enhance non-synaptic GABA release and therefore promote tonic inhibition in HD. To test for this possibility, slices from Q175 WT mice were treated for 10 min with 0.1 mM of D-aspartate (Figure 5A). This had little effect in WT mice (data not shown), but in HD it produced a highly significant increase of I_Tonic(GABA)_ (Figure 5B). In normal mice treatment with 1 mM of D-aspartate produced a significantly higher I_Tonic(GABA)_ if compared to Control (without D-asparate) or treatment with SNAP5115 (Figure 5C), thereby rendering further support to the conclusion that astrocytes indeed release GABA via GAT-3 if glutamate transporter activity is high enough.

Finally, we explored the possibility that the tonic GABA(B)-mediated depression of synaptic GABA release is also influenced by glutamate transporter activity. The experiments were performed in slices from R6/2 HD mice (Figures 5D–F). In one and the same cell, synaptic transmission was sequentially tested under control condition, after 5 min of bath application of D-aspartate (1 mM) and 1 mM of D-aspartate supplemented with the GABA(B) receptor blocker CGP (1 μM). It can be seen that D-aspartate reduced the amplitude of eIPSCs and increased the PPR. This effect was prevented by CGP, supporting a GABA(B)-dependent mechanism that is maintained by astrocytic GABA release via GLT-1/GAT-3 clusters.

The results with D-aspartate are interesting because they show that stimulation of glutamate uptake may restore astrocytic GABA release and therefore help to repair the imbalance between extracellular glutamate and GABA levels in HD. They also suggest that in HD, extrasynaptic GABA(A) and presynaptic GABA(B) receptors are present at sufficient densities to obtain a functional recovery within few minutes.

### Rescue of I_Tonic(GABA)_ with THIP (gaboxadol)

**Figure 6:**
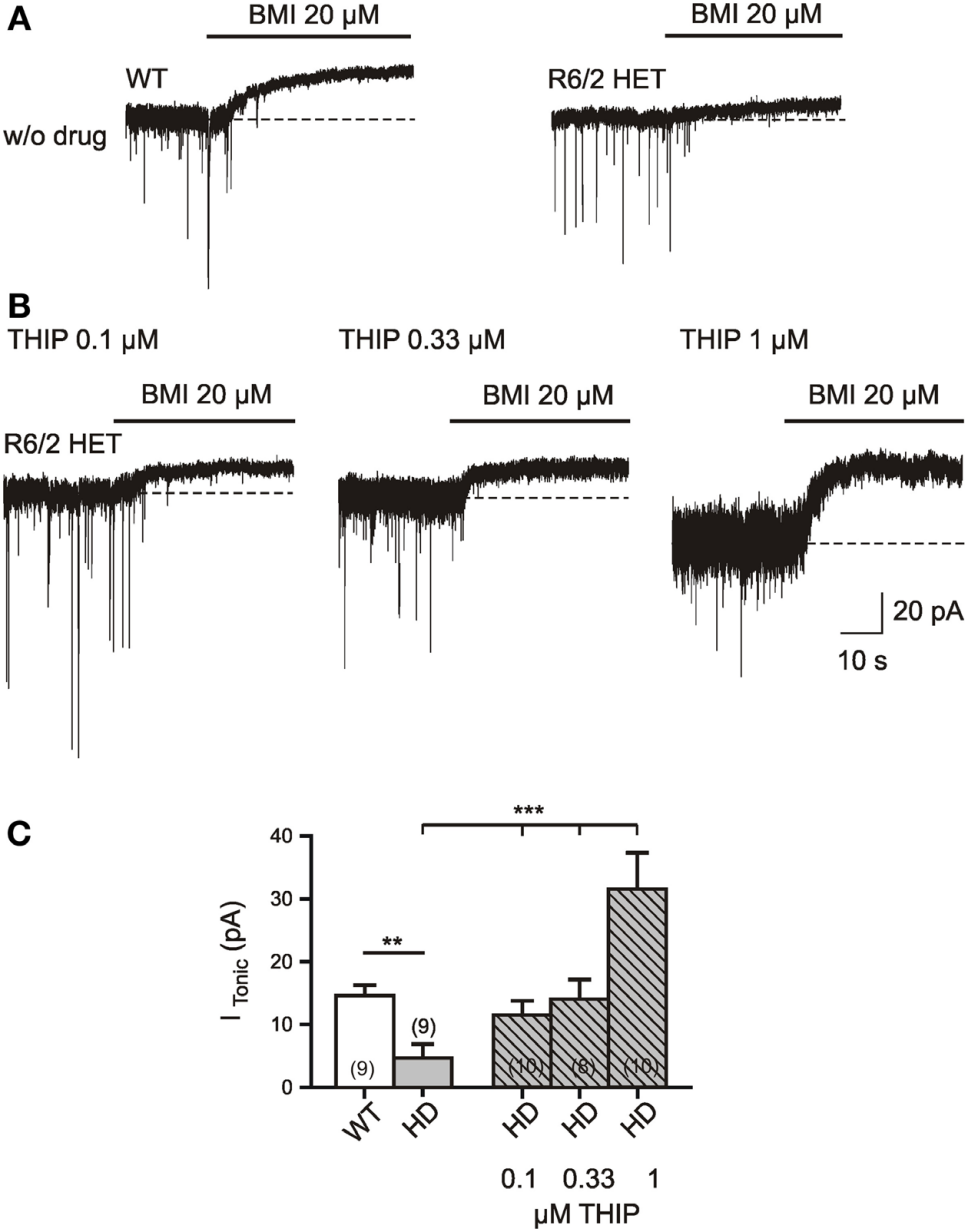
**Rescue of I_Tonic(GABA)_ in R6/2 HD mice by bath application of THIP (gaboxadol) at a concentration of 0.33 μM. (A,B)** Specimen records in the absence **(A)** and presence **(B)** of delta subunit specific GABA(A) receptor hyper-agonist THIP (0.1 μM, 0.33 μM, and 1 μM). The recording conditions were similar to those in Figure 1. Experiments were performed in the presence of TTX (1 μM), DNQX (10 μM), APV (50 μM), LY341495 (40 μM); no K channel block; *V*_*h*_ = −70 mV, *E*_Cl_ = 4 mV. THIP was applied through a slow drug delivery system to the bath solution (exchange time ca. 1 min). **(C)** Quantification of the results. The asterisks denote the following: ^*^p < 0.05, ^**^p < 0.01, and ^***^p < 0.001. The comparison between WT and HD was performed by the Mann–Whitney test. The THIP effect on HD SONs was tested by One-Way ANOVA (multiple comparison against the “THIP0” group) and resulted in *F*_(3, 33)_ = 9.731, *p* < 0.0001 for the THIP concentrations 0, 0.1, 0.33, and 1 μM.

Pharmacological rescue of I_Tonic(GABA)_ is important, but it might be advantageous to achieve its recovery without a reduction of synaptic GABA release. We therefore examined the effects of the delta-subunit-specific GABA(A) receptor hyperagonist THIP (Figures 6A,B). The latter was applied at least 5 min prior to addition of BMI. The records were performed in the presence of TTX, DNQX, APV, and LY341495. It was found that in HD about 0.33 μM of THIP were sufficient to restore I_Tonic(GABA)_ to the WT level (Figure 6C). As it cannot be excluded that THIP at higher concentrations activated both extrasynaptic and synaptic GABA(A) receptors, we estimated the average amplitudes of miniature IPSCs expecting an IPSC decrease if part of the receptors were occupied by THIP. However, no significant difference was found between the IPSC amplitudes recorded in the absence of THIP and THIP at a concentration of 0.33 μM. Thus, application of THIP might be another means to rescue tonic inhibition in the striatum afflicted by HD.

## Discussion

Our experiments demonstrate that symptomatic HD is associated with a weakness of tonic GABA action and illuminate a so far neglected pathophysiological element of HD in astrocytes, the incapacity of GAT-3 to release GABA under condition of astrocyte depolarization. The weakness of non-synaptic GABA release from astrocytes in HD was not only reflected by the lower amplitudes of I_Tonic(GABA)_ but also by the reduced strength of GABA(B)-mediated presynaptic depression of synaptic GABA release. Interestingly, treatment of slices with an exogenous substrate of glutamate transport rescued non-synaptic GABA release supporting the hypothesis that GAT-3 activity is coupled to astrocytic glutamate transport (Heja et al., 2009, 2012).

### Reduced extracellular GABA concentration in HD?

Several attempts have been made to determine transmitter concentrations in postmortem normal and HD brains and by dialysis and HPLC of animal preparations. A pioneer study by Spokes et al. (1979) reported an unchanged GABA concentration in the postmortem HD striatum despite a significant decrease in the activity of glutamate decarboxylase. Later studies described a massive decrease of [GABA]_ec_ in the striatum (Ellison et al., 1987) and globus pallidus of choreic patients (Kanazawa et al., 1985) and in mice treated with quinolinic acid, an earlier model of HD (Ellison et al., 1987). However, to measure ambient transmitter concentrations under resting conditions and independently of synaptic GABA release, it might be better to take advantage of endogenous high affinity receptors (Le Feuvre et al., 1997; Dvorzhak et al., 2010), unless the transmitter receptors themselves set limits to the detection of extracellular transmitters. This was not the case here, since in HD slices with GAT-1 block or treatment with D-aspartate I_Tonic(GABA)_ amplitudes were even higher than in the respective WT controls.

Nonetheless, it would be premature to exclude the possibility that cell-type-specific differences/changes in the expression of transmitter receptors add to the presently observed HD-related differences. It is known that in the adult brain, I_Tonic(GABA)_ primarily depends on GABA(A) receptors containing a delta subunit (Stell et al., 2003). The latter is likely to be more abundant in D1-expressing SONs (Santhakumar et al., 2010). Indeed, in the absence of TTX, HD-related differences might be more prominent in D2 SONs (Cepeda et al., 2013).

### Astrocytic GABA release?

In the rodent striatum 95% of the neurons express a GABAergic phenotype, and interneurons together with other SONs establish an extensive coverage with GABAergic synapses (Wilson, 2007; Tepper et al., 2008). Striatal slices of WT and HD origin exhibit AP-dependent spontaneous activity (Dvorzhak et al., 2013). It is therefore not surprising that a major part of I_Tonic(GABA)_ depended on AP-mediated synaptic release of GABA. The present experiments showed, however that in both HD models the difference between WT and HD persisted after exclusion of AP-dependent synaptic GABA release with TTX.

GABA of non-synaptic origin might be provided through the GABA transporters GAT-1 and GAT-3 [see Jin et al. (2011a) for a recent comprehensive review on data from basal ganglia]. However, previous results from the normal rodent striatum suggested an increase of [GABA]_ec_ after pharmacological block of GAT-1 (Waldmeier et al., 1992; Kirmse et al., 2008). This result is now confirmed and extended by showing that the NO-711-induced increase of GABA uptake was even larger in HD.

The remaining possibility is that GABA is provided by astrocytes. The concept of gliotransmitter release in a voltage- and/or calcium-dependent manner has already received broad support. It is known that astrocytes release amino acids (glutamate, D-serine), nucleotides (adenosine) and neuropeptides (brain-derived neurotrophic factor, natriuretic peptide) and thereby modify the activity of neighboring neurons and their synaptic signals (Parpura and Zorec, 2010). GABA is seldom on the list of gliotransmitters (but see Glykys and Mody, 2007b; Angulo et al., 2008), possibly because astrocytes lack the respective enzymes glutamate decarboxylase, GAD65 and GAD67. However, there is a GAD-independent form of GABA synthesis from putrescine (Seiler et al., 1979; Angulo et al., 2008; Heja et al., 2012). In addition, GABA could also be shuttled from sites of GABA uptake to sites of GABA release, similar to the spatial potassium buffering of glial cells (Lux et al., 1986; Reichenbach, 1991). GABA immunolabel was found in GFAP-positive hippocampal astrocytes (Le Meur et al., 2012).

Previous studies on the role of glial GABA transporters in the rodent brain have yielded somewhat controversial results. Keros and Hablitz (2005) examined the function of GAT-3 and GAT-1 in the juvenile rat cerebral cortex and found that GAT-3 contributes little to the regulation of inhibition unless GAT-1 is blocked with NO-711. In this case, GAT-3 and GAT-1 were thought to operate synergistically in the uptake mode with the prediction that the presence of SNAP-5114 should enhance I_Tonic(GABA)_ as well as GABA(B)-mediated presynaptic depression of evoked IPSCs. Results supporting this notion were obtained in a previous study from our own lab based on recordings from striatal output neurons (SONs) in juvenile normal mice (Kirmse et al., 2009).

Support for a GAT-3-dependent enhancement of inhibition under normal conditions was derived from another study of the rodent cerebral cortex, where recordings were made in layer V pyramidal neurons (Kinney, 2005). In this case, SNAP-5114 was found to increase the amplitude of eIPSCs suggesting a decrease of GABA(B) receptor-mediated depression of synaptic GABA release when GAT-3 is not active. In recordings from globus pallidus neurons, SNAP-5114 was also found to increase eIPSC amplitudes (Jin et al., 2011b).

A recent study on the role of GAT-3 in the hippocampus was performed in freely moving rats using microdialysis with subsequent HPLC measurement of GABA concentration in the acquired probes (Kersante et al., 2013). This study showed a contribution of GAT-3 to GABA uptake when GAT-1 was blocked. No evidence was found for astrocytic GABA release.

As the state of astrocytes in a given slice preparation can have a major impact on the strength of glutamate uptake, on one side, and ambient GABA concentrations, on the other side, it may become necessary to provide some functional checkpoints from astrocytes to compare the results obtained on tonic GABA actions under specific experimental settings.

### Rescue of function in HD

It is known for more than a decade that astrocytes are affected by HD (Vonsattel and DiFiglia, 1998; Faideau et al., 2010). The so far most prominent functional deficit ascribed to astrocytes is the deficiency of glutamate uptake (Lievens et al., 2001; Behrens et al., 2002; Petr et al., 2013) and the resultant elevation of extracellular glutamate concentration in the striatum (Arzberger et al., 1997; Lee et al., 2013).

The present experiments add a new facet to the pathophysiology of HD by demonstrating an HD-related switch in the polarity of GAT-3 action. Our results predict that block of the non-physiological GABA uptake in HD would, within few minutes, restore the characteristics of tonic GABA actions to WT level. SNAP5114 is a nipecotic acid derivative with “moderate affinity and selectivity for the cloned human GAT-3” (Dhar et al., 1994). It has an IC_50_ of about 5 μM for GAT-3, 20 μM for GAT-2, 140 μM for BGT-1, and 400 μM for GAT-1. Should more effective and selective blockers of GAT-3 become available, it might become worthwhile to examine the capacity of GAT-3 to alleviate motor symptoms of HD.

At present, the easiest way to restore I_Tonic(GABA)_ seems to be the application of gaboxadol (THIP). This compound is an already well-characterized delta-subunit-specific GABA(A) hyperagonist and has for some time been considered for treatment of insomnia (Wafford and Ebert, 2006). In view of the presently discovered HD-related deficit in I_Tonic(GABA),_ the question arises whether HD patients can benefit from this drug. Unfortunately, in a short (2 weeks) clinical trial on four patients with choreatic and one patient with hypokinetic-rigid type of HD, gaboxadol failed to provide the expected benefits but merely caused sleepiness and unsteadiness of gait (Foster et al., 1983).

Finally, our results with D-aspartate underscore the need to stimulate/recover the function of glutamate transport. A promising agent along this line is the antibiotic ceftriaxone (Sari et al., 2010). Further experiments will show to what extent this drug can restore tonic GABA actions in case of symptomatic HD.

### Conflict of interest statement

The authors declare that the research was conducted in the absence of any commercial or financial relationships that could be construed as a potential conflict of interest.
